# Disseminated *Acanthamoeba castellanii* infection in a patient with AIDS: a case report and literature review

**DOI:** 10.3389/fmed.2024.1377302

**Published:** 2024-06-17

**Authors:** Qunqun Jiang, Zhongwei Zhang, Yuxiang Cai, Liangjun Chen, Liping Deng, Yong Xiong

**Affiliations:** ^1^Department of Infectious Disease, Zhongnan Hospital of Wuhan University, Wuhan, China; ^2^Department of Pathology, Zhongnan Hospital of Wuhan University, Wuhan, China; ^3^Department of Laboratory Medicine, Zhongnan Hospital of Wuhan University, Wuhan, China

**Keywords:** *Acanthamoeba castellanii*, AIDS, diagnosis, metagenomic next-generation sequencing, treatment, case report

## Abstract

**Background:**

*Acanthamoeba castellanii* infection is a rare condition primarily occurring in immunocompromised patients with extremely high mortality. Currently, there is no standard treatment for this condition, and successful treatment reports are scarce.

**Case presentation:**

We present a case of *Acanthamoeba castellanii* infection in a 63-year-old female patient with AIDS, who was admitted to our hospital with symptoms of fever, skin ulcers, subcutaneous nodules, and food regurgitation from the nose while eating. After initial empirical treatment failed, a biopsy of the subcutaneous nodule was performed, and metagenomic next-generation sequencing (mNGS) technology was used to detect pathogenic microorganisms in both the biopsy specimen and blood samples. The results revealed *Acanthamoeba castellanii* infection. Additionally, histopathological examination of the biopsy specimen and cytological examination of the secretions from the ulcer surface also confirmed this pathogenic infection. The patient’s symptoms significantly improved upon discharge after adjusting the treatment regimen to a combination of anti-amebic therapy.

**Conclusion:**

Immunocompromised patients presenting with unexplained fever and skin or sinus lesions should be evaluated for *Acanthamoeba castellanii* infection. Multi-drug combination therapy is required for this organism infection, and a standard treatment protocol still needs further research. Metagenomic next-generation sequencing is a valuable tool for early diagnosis of unknown pathogen infections.

## Introduction

*Acanthamoeba castellanii* is a ubiquitous, free-living protozoan that infrequently causes disease in humans. Infections by this organism predominantly affect immunocompromised individuals and are often associated with a grave prognosis (1, 2). *Acanthamoeba castellanii* can infect various organs throughout the body, leading to disseminated infections and even central nervous system infections. The clinical presentation is varied and non-specific, posing a significant challenge for timely diagnosis by clinicians. Furthermore, there is currently no established treatment for this pathogenic infection, and successful treatment reports are scant (3, 4). This report presents a case of *Acanthamoeba castellanii* infection in an AIDS patient and reviews the literature on successful treatment of this pathogen infection.

## Case presentation

A 63-year-old female patient was admitted to our department with symptoms of fever, skin ulcers, subcutaneous nodules, and food regurgitation from the nose while eating. The patient is a farmer living in a rural area who was diagnosed with AIDS 2 years ago and started receiving antiretroviral therapy (ART). However, she did not take the medications regularly and has been completely off them for several months. Four months ago, the patient experienced oral pain and found a purulent nodule on the upper palate. One month later, the nodule on the upper palate ruptured, forming a sinus that communicated with the nasal cavity, and the patient experienced nasal food regurgitation while eating. In addition, the patient had intermittent fever and multiple subcutaneous nodules on the trunk and limbs. The subcutaneous nodules gradually grew and ruptured, leading to non-healing ulcers. Throughout this process, the patient did not experience symptoms such as cough, headache, abdominal pain, diarrhea, vision loss, visual field defect, or limb mobility disorders.

**Figure 1:**
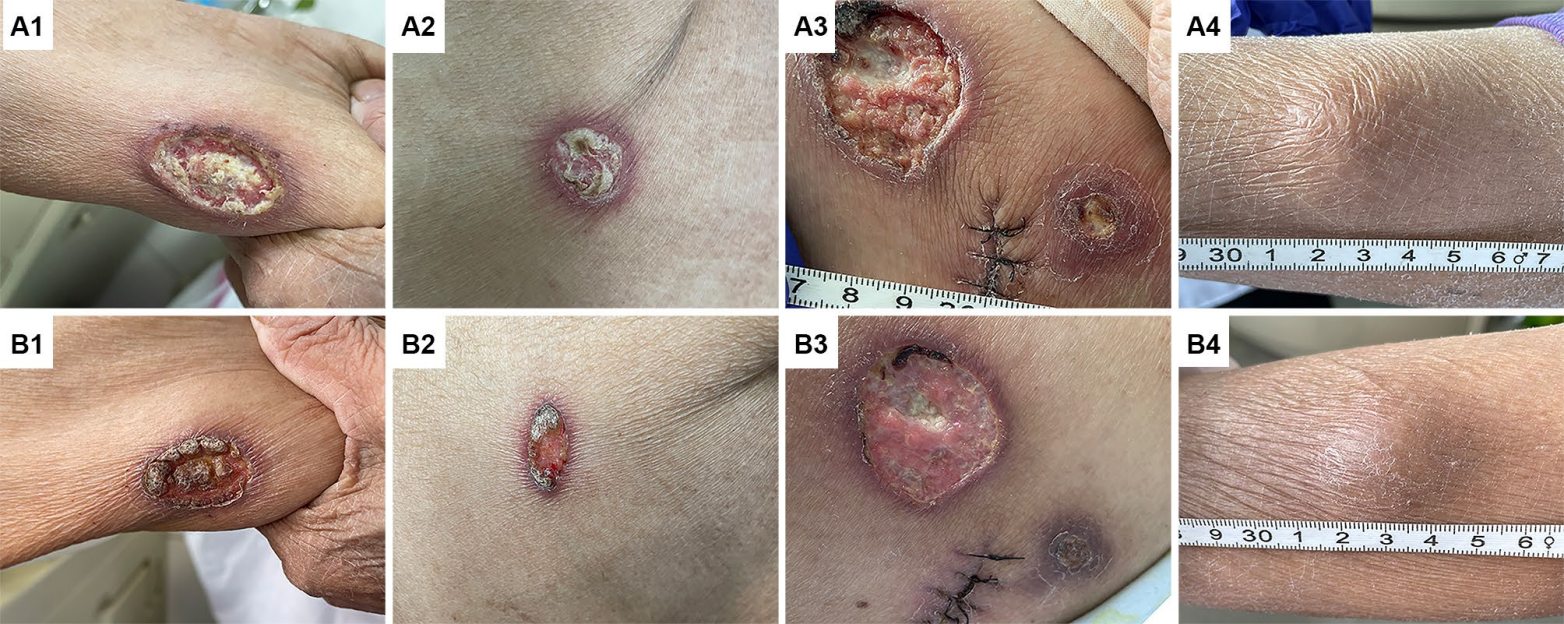
Changes in skin lesions before and after anti-amoeba treatment. Images **(A1–A4)** show the ulcers of the right upper limb **(A1)**, the right lateral chest wall **(A2)**, the right hip **(A3)**, and the subcutaneous nodule of the right lower leg **(A4)** before treatment, respectively. These lesions were significantly improved after anti-amoeba treatment **(B1–B4)**.

**Figure 2:**
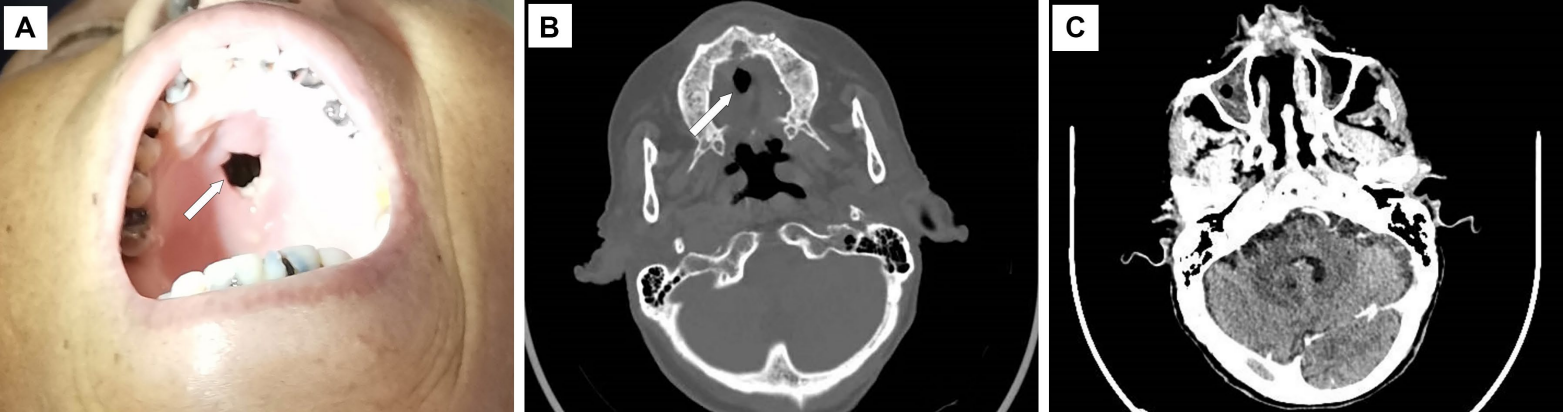
Upper palate defect and maxillofacial CT scan image. There is a defect of approximately 1 cm in diameter on the upper palate **(A, B)**, and the CT scan shows the thickening and enhancement of the nasal mucosa **(C)**. The white arrow indicates the defect.

Upon admission, the physical examination revealed several ulcers and subcutaneous nodules on the trunk and limbs. The largest area of the ulcer was approximately 5*4cm^2^ and covered with necrotic tissue and purulent drainage. Subcutaneous nodules had mild tenderness and were poorly demarcated from surrounding tissues (Figure 1). The patient’s oral mucosa had leukoplakia and a sinus tract with a diameter of approximately 1 cm in the upper palate that communicated with the nasal cavity (Figure 2A). There was also a small, unruptured, purulent nodule next to it. The patient’s neck was soft, her vision was normal, and the superficial lymph nodes were not enlarged.

On admission, laboratory examination revealed a white blood cell count of 2.3 × 10^9^/L, a hemoglobin level of 74 g/L, normal liver and kidney functions except for low albumin levels (28.7 g/L), a normal procalcitonin level, a C-reactive protein level of 21.7 mg/L, and a red blood cell sedimentation rate of 27 mm/h. The CD4+ T-lymphocyte count was 4 cells/μl, and the HIV-RNA viral load was 94,000 copies/ml. Serological studies for (1, 3) β-D-glucan, galactomannan (GM), and interferon-gamma release assay (IGRA) were negative. Blood screening for syphilis, cytomegalovirus, and Epstein–Barr virus was also negative. Maxillofacial computed tomography (CT) scan demonstrated thickening and enhancement of the sinus mucosa and partial tissue defects in the hard palate and nasal septum (Figures 2B,C). A chest CT scan revealed a mild infection in the left lung. Color Doppler ultrasonography of the right lower extremity nodule revealed a hypoechoic area measuring 4.0 × 2.8 × 1.7 cm located at 0.20 cm under the skin with an unclear outline, irregular shape, uneven echogenic areas, and small anechoic patches inside, which was considered an inflammatory mass.

**Figure 3:**
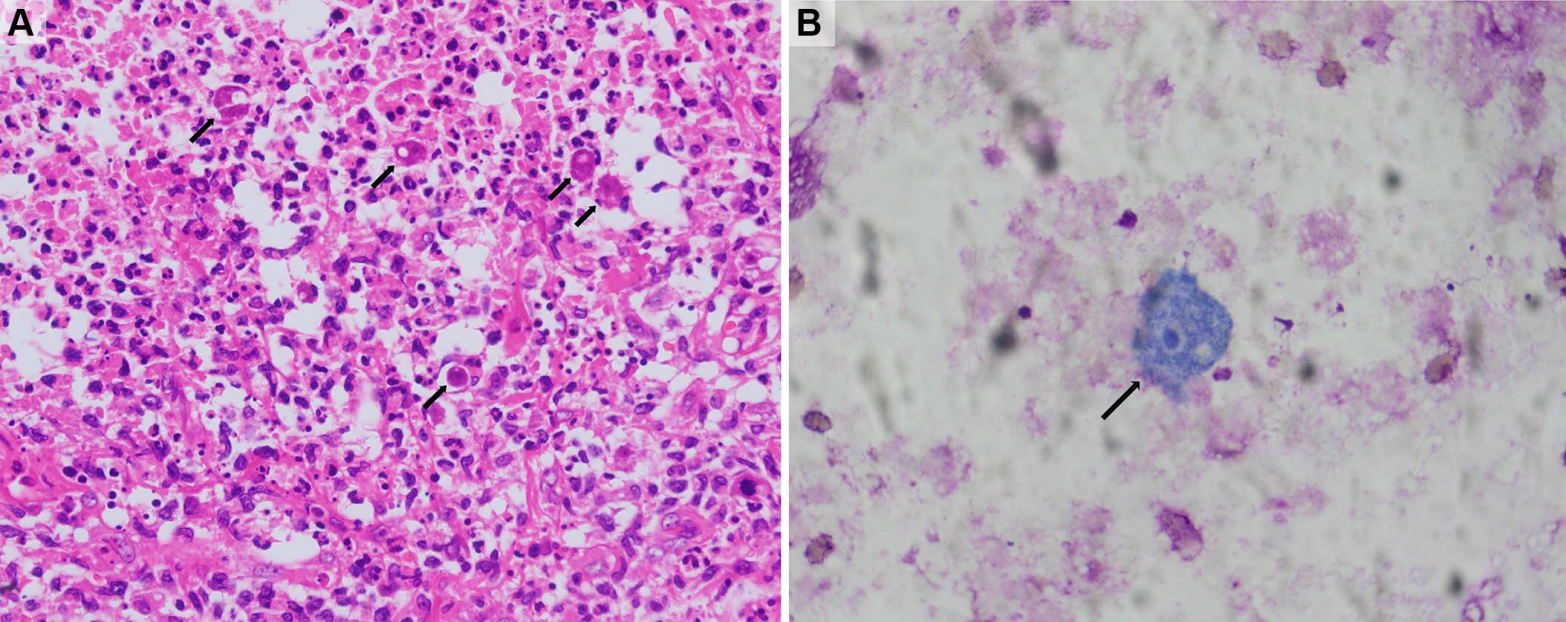
Histology and cytology findings. **(A)** The histological study of subcutaneous mass in the right hip showed granulomatous inflammation and amoeba trophozoites (hematoxylin and eosin, 200×). **(B)** The cytological study of ulcer surface secretion showed amoeba trophozoite (Wright-Giemsa stain, 400×). The black arrows indicate amoeba trophozoites.

The patient initially received treatment with fluconazole (200 mg daily), cefotaxime/sulbactam sodium (3.0 g q12h), and trimethoprim-sulfamethoxazole (TMP/SMZ) (0.96 g daily) to cover common bacterial and fungal infections. However, the patient’s symptoms did not improve. On the second day of admission, the patient began experiencing recurrent high fevers with a temperature of over 39°C. The skin ulcers showed no signs of healing, and the subcutaneous nodules did not shrink. On the third day, a biopsy of the right hip subcutaneous mass was performed. Histopathological analysis, metagenomic next-generation sequencing (mNGS) testing, and special staining of pathogenic microorganisms were carried out. Peripheral blood was collected for pathogenic microorganism mNGS at the same time. Additionally, cytological examinations of ulcer surface secretions and sinus secretions were performed. On the fifth day, the mNGS results of both tissue and peripheral blood revealed *Acanthamoeba castellanii* infection (The gene sequence has been deposited in GenBank under accession number PRJNA1108617). Pathological analysis of the biopsy tissue showed chronic suppurative inflammation with abscess formation, partial tissue degeneration and necrosis, and local granulomatous inflammation with multinucleated giant cell reaction. Trophozoite-like structures were observed, which are considered to be indicative of an amoebic infection (Figure 3A). Tissue periodic acid-Schiff staining, Gram staining, silver staining, and acid-fast staining were all negative. Cytological examination of ulcer surface secretion also found amoebic trophozoite (Figure 3B). Blood cultures were reported as negative. Later, the patient underwent a head MRI, cerebrospinal fluid (CSF) pressure measurement, and ophthalmological examinations. No visible abnormalities were found in the head MRI; CSF pressure and leukocyte count were normal; and glucose and protein levels were slightly elevated (glucose concentration of 4.85 mmol/L and protein concentration of 0.67 g/L). *Cryptococcus neoformans* capsule antigen, India ink stain, acid-fast stain, and polymerase chain reaction (PCR) for herpes simplex virus, cytomegalovirus, and Epstein–Barr virus were all negative in the CSF. Bacterial and fungal cultures and cytological examination of the CSF were also negative. No retinal or corneal diseases were found during ophthalmological examination.

The patient presented with high fever, skin nodules, ulcers, and the formation of oral sinuses. Blood and tissue pathogen microorganism mNGS, histopathology, and cytology of ulcer exudate all detected *Acanthamoeba castellanii*, while other tests for bacteria, tuberculosis, fungi, and virus-related tests did not find any other pathogenic microorganisms. Based on the patient’s clinical symptoms and test results, we considered the possibility of disseminated *Acanthamoeba castellanii* infection. Subsequently, we adjusted the treatment according to literature reports, choosing a combination therapy of voriconazole injection (0.2 g q12h), metronidazole injection (0.5 g q8h), and TMP/SMZ (1.44 g q8h). At the same time, local cleaning and disinfection of the ulcers were performed with the external application of ketoconazole cream. From the eighth day onward, the patient did not present fever again, the skin ulcers began to heal, and the subcutaneous nodules decreased in size. On hospital day 11, the patient restarted combined antiretroviral therapy (cART, lamivudine/tenofovir disoproxil fumarate/dolutegravir) and was discharged on the 15th day of hospitalization. On discharge, the patient was advised to continue itraconazole and compound sulfamethoxazole tablets for maintenance treatment. Three months after discharge, we conducted a telephone follow-up, but unfortunately, the patient had passed away. The family of the patient indicated that after leaving the hospital, the patient did not continue taking medication as advised and developed a new infection, which led to the worsening of the condition. The patient refused to be hospitalized for treatment again, which ultimately led to the patient’s death.

## Discussion and conclusion

In this article, we present a case of *Acanthamoeba castellanii* infection in an AIDS patient, which is the first confirmed case of Acanthamoeba infection in our center. *Acanthamoeba castellanii* is a free-living organism distributed worldwide and has been isolated from diverse environmental sources, including soil, sewage, air pipes, and various other common locations (1, 3). Although most people have been exposed to Acanthamoeba and have serum antibodies against it, Acanthamoebiasis is an extremely rare clinical condition that usually occurs in patients with AIDS or patients undergoing immunosuppressive therapy for organ transplantation or immunological diseases without HIV infection (1, 5).

*Acanthamoeba castellanii* infection is highly lethal; disseminated acanthamoebiasis without central nervous system infection is reported to have a mortality rate of approximately 73%, while mortality in patients with central nervous system infection exceeds 90% (6). Early diagnosis and prompt treatment are critical for improving patient outcomes but can be challenging for clinicians. Depending on the host immunity status, *Acanthamoeba castellanii* infection can spread to the skin, lungs, eyes, central nervous system, and other organs. Clinical manifestations vary depending on the site of infection and can include fever, skin nodules or ulcers, sinus lesions, headaches, convulsions, neurological dysfunction, and other symptoms commonly seen in infections caused by bacteria, fungi, mycobacteria, and parasites (3, 6, 7). Moreover, *Acanthamoeba castellanii* infection is rare in the clinic, and most clinicians are unfamiliar with the disease, making it easy to misdiagnose. Patients may require repeated visits to receive a diagnosis and cannot be treated in a timely manner (1, 8). For patients with AIDS and skin and sinus lesions, in addition to considering possible diagnoses of common pathogen infections, *Acanthamoeba* infection should also be considered as a possible differential diagnosis.

The traditional method for diagnosing *Acanthamoeba* infection primarily relies on histological examination and microbial culture (1, 3). The typical histopathological manifestation of acanthamoebiasis is granulomatous inflammation, and the presence of amoebic trophozoites or cysts in pathological tissue or culture specimens can confirm infection with the pathogen. However, in severely immunocompromised patients, histological findings may be atypical, and amebic trophozoites and cysts may be easily mistaken for reactive histiocytes or yeasts, which is highly dependent on the experience of the pathologist (9). Polymerase chain reaction (PCR) and serological tests are also commonly used for diagnosis, but careful consideration is required before testing. mNGS is a new diagnostic technology that can sequence the entire microbial genome in a specimen, providing high sensitivity and efficiency in pathogen detection (10, 11). In recent years, more and more patients with unexplained infections have applied mNGS for timely diagnosis, which has greatly aided in their diagnosis; our patient also benefited from the prompt diagnosis enabled by mNGS. However, mNGS is susceptible to environmental and human factors, so caution must be exercised when interpreting reports in conjunction with the patient’s condition.

Currently, there is no established treatment for *Acanthamoeba* infection, and successful treatment cases are rare. Many therapeutic agents have been tested *in vitro* for their ability to inhibit the activity of pathogenic *Acanthamoeba*, but the results are conflicting, and there is no direct evidence that these drugs are effective in individuals. Taravaud et al. evaluated 15 drugs for their *in vitro* activity on *Acanthamoeba castellanii* and found that hexamidine, voriconazole, and clotrimazole had the highest activity, while rifampicin, metronidazole, and cotrimoxazole were inactive, and amphotericin B activity increased with time of use (12). The Infectious Diseases Society of America (IDSA) guidelines recommend trimethoprim-sulfamethoxazole, pyrimethamine, sulfadiazine, rifampicin, and ketoconazole or fluconazole for the treatment of granulomatous encephalitis caused by *Acanthamoeba* infection. However, the supporting evidence is of low quality (Level C—IDSA-US Public Health Services Grading System III) (13). Miltefosine is an anti-cancer drug that is increasingly used in the treatment of *Acanthamoeba* infections, and reports have shown that it may be beneficial to reduce mortality, but it has not been formally included in the drug indications (14, 15).

In this report, we summarized the cases of successful treatment for disseminated *Acanthamoeba castellanii* published on PubMed (Table 1) (8, 14, 16–29). The most commonly used drugs in these successful cases include TMP/SMZ, fluconazole, itraconazole, rifampicin, and miltefosine. These reports suggest that combination therapy is often more effective than monotherapy, but the results of combination therapy can also be inconsistent. Teh and colleagues reported a case of chronic lymphoma with disseminated cutaneous *Acanthamoeba castellanii* infection. The patient was successfully treated with a combination of pentamidine, miltefosine, TMP/SMZ, posaconazole, and azithromycin (27). In contrast, Damhorst et al. reported a case of *Acanthamoeba castellanii* meningitis in an AIDS patient. The patient was treated with a combination of miltefosine, flucytosine, pentamidine, sulfadiazine, fluconazole, and azithromycin. Unfortunately, the patient did not respond to the treatment and passed away (2). In this case, we opted for a combination treatment that included intravenous voriconazole and metronidazole, oral trimethoprim/sulfamethoxazole, and topical ketoconazole cream. Furthermore, previous studies have suggested that early initiation of antiretroviral therapy may enhance the prognosis of HIV-positive patients (2, 24). We also promptly commenced antiretroviral therapy as part of the comprehensive treatment plan for our patient. Following the treatment, the patient’s symptoms showed significant improvement. Although our treatment regimen has shown certain effects in this patient, further research is still needed to establish a standardized protocol for treating *Acanthamoeba castellanii* infection.

**Table 1:** Published reports of successfully treated disseminated *Acanthamoeba castellanii* infection.

Year	Age/gender	Region	Underling conditions	Lesion involved sites	CD4 count (cells per μL)	Treatment	Citation
Immunocompromised patients							
1993	33/M	America	AIDS, hemophilia	Skin, Sinus	0	Ketoconazole (200 mg q8h), 5-Fluorocytosine (40 mg/kg q8h)	Helton et al.
1999	39/F	America	Lung transplant	Skin, Lung, Liver	NA	Itraconazole, pentamidine, 5-flucytosine, topical chlorhexidine gluconate, and ketoconazole	Oliva et al.^#^
2000	33/M	Spain	AIDS	CNS	82	Sulfadiazine, pyrimethamine, fluconazole, lesion resection	Martinez et al.^#^
2000	37/M	America	AIDS, non-Hodgkin’s lymphoma	Sinus, Skin	NA	Pentamidine (4 mg/kg/d), fluorocytosine (75 mg/kg/d), sinuses debridement, Itraconazole (200 mg qd); topical chlorhexidine, ketoconazole;	Teknos et al.
2001	35/F	America	AIDS	Sinus, Skin	76	Surgical debridement, itraconazole, azithromycin, 5-flucytosine, and rifampin	Rosenberg et al.^#^
2002	35/F	America	AIDS	Sinus, Skin	50	sinuses debridement, pentamidine(4 mg/Kg/d), levofloxacin (500 mg qd), fluorocytosine (2 g q6h), amphotericin B (550 mg qd); itraconazole (200 mg bid)	Rivera, et al
2008	25/M	America	*Mycobacterium tuberculosis*	CNS, Lung, Skin	182	TMP/SMZ, fluconazole, Miltefosine	Aichelburg et al.^#^
2011	25/M	Austria	Acute lymphoblastic leukemia	CNS	NA	TMP/SMZ (6 mg/kg/d), fluconazole (10 mg/kg/d), pentamidine (4 mg/kg/d), and miltefosine (2.5 mg/kg/d)	Maritschnegg et al.
2017	38/M	America	AIDS	CNS	19	Miltefosine, fluconazole, cotrimoxazole, flucytosine	Sahly et al.^#^
2022	70 M	Australia	Chronic lymphocytic leukemia	Skin	NA	pentamidine (280 mg qd), miltefosine (50 mg, q8h). TMP/SMZ, posaconazole; azithromycin (500 mg, qd)	Teh et al.
Immunocompetent patients							
2001	8/M	India	Immunocompetent	CNS	NA	TMP/SMZ (20 mg/kg/d), ketoconazole(5 mg/kg/d) rifampin(10 mg/kg/d)	Singhal et al.
2001	3/M	India	Immunocompetent	CNS	NA	TMP/SMZ (20 mg/kg/d), Ketoconazole (5 mg/kg/d), rifampin (10 mg/kg/d)	Singhal et al.
2002	45/F	India	Immunocompetent	CNS	NA	Rifampin (600 mg qd), TMP/SMZ (960 mg bid), fluconazole (200 mg qd), albendazole (200 mg bid) and ceftriaxone (2 g q12h);	Hamide et al.
2009	63/M	Taiwan	Immunocompetent	CNS	NA	Amphotericin B, rifampin, corticosteroids	Sheng et al.^#^
2012	3/M	India	Malnourished	CNS	NA	TMP/SMZ(20 mg/kg/d), rifampicin (10 mg/kg/d), ketoconazole (5 mg/kg/d),	Khurana et al.
2012	38/M	Canada	Immunocompetent	CNS	NA	Voriconazole (100 mg bid), miltefosine (100 mg bid)	Webster et al.
2014	30/M	India	Immunocompetent	CNS	NA	Rifampicin (600 mg qd), Cotrimoxazole (960 mg bid), Fluconazole (400 mg qd)	Khanna et al.

^#^Therapeutic drug dosage is not mentioned.

TMP/SMZ, trimethoprim/sulfamethoxazole; CNS, central nervous system; NA, not available.

In conclusion, we have reported a case of *Acanthamoeba castellanii* infection in an AIDS patient. This case highlights the importance of considering *Acanthamoeba castellanii* infection in immunocompromised patients with unexplained fever and skin or sinus lesions. Early diagnosis and prompt treatment are crucial for improving prognosis, and further research is needed to establish a standardized treatment protocol. mNGS is a promising tool for the early diagnosis of unknown pathogen infections, particularly those caused by rare pathogens.

## Data availability statement

The raw data supporting the conclusions of this article will be made available by the authors, without undue reservation.

## Ethics statement

This study was conducted according to the principles expressed in the Declaration of Helsinki and was approved by the ethics committee of Zhongnan Hospital of Wuhan University. Written informed consent was obtained from the individual(s) for the publication of any potentially identifiable images or data included in this article. Written informed consent was obtained from the participant/patient(s) for the publication of this case report.

## Author contributions

QJ: Writing – original draft, Writing – review & editing. ZZ: Writing – original draft, Writing – review & editing. YC: Data curation, Writing – review & editing. LC: Data curation, Writing – review & editing. LD: Writing – review & editing, Project administration. YX: Writing – review & editing, Project administration.

## Supplementary material

Supplementary FIGURE S1 Timeline of the case report. TPM/SMZ: Trimethoprim/sulfamethoxazole; cART: combined antiretroviral therapy.
