# Supplementary figures and images for: Disseminated Acanthamoeba castellanii infection in a patient with AIDS: a case report and literature review
Source: Front Med (Lausanne). 2024 Jun 17;11:1377302. doi: 10.3389/fmed.2024.1377302 (PMC11215114; doi:10.3389/fmed.2024.1377302)

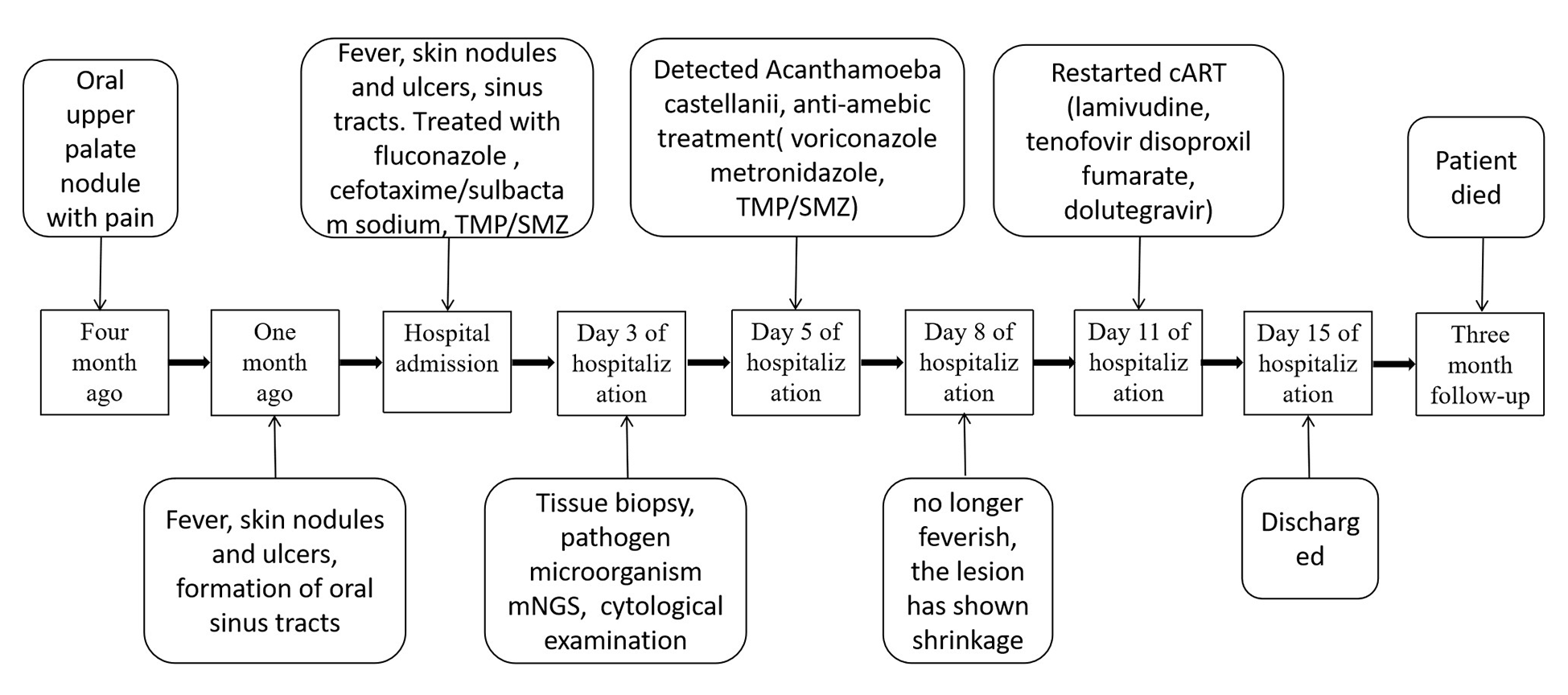

Supplement: Supplementary FIGURE S1 — Timeline of the case report. TPM/SMZ: Trimethoprim/sulfamethoxazole; cART: combined antiretroviral therapy. [file Image_1.TIF]
